# 

**Published:** 1893-05-27

**Authors:** 


**OUR NEW OFFICES.**
428, Strand, W.C., will henceforth be the Office of this Journal.

**Notices of Births, Deaths, and Marriages,** are inserted in this column at a charge of 2s. 6d. for an announcement not exceeding four lines.

**MARRIAGE.**

HODGSON—STEEL.—At St Mary's, Bootle, Liverpool, on the 24th inst., by the Rev. J. Bullen, Vicar, John Hodgson, of South View, Gainford (late of North Terrace, Darlington), to Lucy J. Steel, of Liverpool.

### NOTICE TO CORRESPONDENTS.

All MS., Letters, Books for Review, and other matters intended for the Editor should be addressed THE EDITOR, THE LODGE, PORCHESTER SQUARE, LONDON, W.

The Editor cannot undertake to return rejected M.S., even when accompanied by stamped directed envelope.

**Notice to Advertisers and Subscribers.**

All Advertisements, Orders for Copies of THE HOSPITAL, and Business Communications should be addressed to The Publisher, *not to the Editor*, THE HOSPITAL, 428, Strand, W.C.

The Cost of Subscription, post free, is as follows:—Per week, 2½d.; per quarter, 2s. 6d.; per half-year, 5s.; per annum, 8s. 6d. Foreign:—Per annum, 10s. 8d.; payable, in all cases, in advance.

**"Burdett's Hospital Annual," 1893.**

Fourth year of publication. 700 pp. Boards, gilt lettering. A most complete guide to British, Colonial, Indian, and American Hospitals, Dispensaries, Nursing and Convalescent Institutions, and Asylums. Price 5s. Published by THE SCIENTIFIC PRESS (Limited), 423, Strand, W.C.

**NOTICE.—The Index for Vol. XIII. (ending March 1893) is on sale at the Office of this Journal, price 2½d., post free.**

### SCRAPS AND GLEANINGS.

A NEW infectious diseases hospital is to be shortly erected at Paignton.

SIR WILLIAM CUNLIFFE-BROOKS has benefited the Manchester Royal Infirmary with a generous gift of £1,000.

THE Barnsley Hospital at Richmond shows at the annual statement of accounts that the net receipts for 1892 were £1,415.

THE Municipal Council of Paris have decided that they will name certain streets in their capital, after celebrated physicians

THE annual meeting of the Exeter Port Sanitary Authority was held lately whereat a discussion was raised as to the proposed hospital for the Port.

THE matinée held by Mr. Basil Broke in aid of the Hospital for Diseases of the Throat, Golden Square, has resulted in a gain of £155 to the institution.

IT is proposed to erect a convalescent home at Inverness in connection with the Northern Infirmary. The funds already collected for the scheme are £154.

MONS. M. LOIR, nephew and pupil of Mons. Pasteur, has been nominated director of the new Bacteriological Institute about to be established in Sydney.

A GREAT Kosmic fête and bazaar was held on May 16th at the Royal Dublin Society's premises at Balls Bridge, in aid of the funds of the City of Dublin Hospital.

BARON ROTHSCHILD has contributed a large sum of money towards erecting an institute at Winer Wald in Austria, for the climatic treatment of Tuberculosis.

THE recently-issued report of the Jewish Board of Guardians states that out of the 30,000 applications for relief received by the Board, 95 per cent. received substantial support.

THE East End Tradesmen's Association, in aid of the London Hospital, has, during the 21 years of its existence, contributed about £2,600, in addition to providing 80 life-governors.

WITH a view to assisting the funds of the Cold Ash Cottage Hospital for Children, it is proposed to hold a floral fête in July, at which the children of the various schools are invited to assist.

THE residents of Lewisham have learnt with regret that the Metropolitan Asylums Board have decided to purchase the Wilderness estate at Hither Green for the erection of the proposed fever hospital.

SEVERE epidemics of influenza and scarlatina are recorded from Strassburg. The losses have been amongst the aged principally in the case of the first disease and amongst the very young in the second.

WITH a view of helping to raise money for the purpose of paying for the renovation of Addenbrooke's Hospital an enormous jumble sale was held in the Corn Exchange at Cambridge in the first week of May.

THE Wolverhampton General Hospital Ambulance Van rendered most important and efficient service in connection with the late accident at the Staffordshire Steel and Ingot Fire Company's works, Spring Vale.

THE children of the London poor have derived great benefit by the gymnasium at Victoria Park which the County Council have laid out at a cost of £750. This gymnasium covers an area of 3,000 square yards.

THE finances during the year have proved very satisfactory with regard to the funds of the Nottingham and Midland Eye Infirmary. There has been also an improvement in the number of the annual subscriptions.

WITH a view to furnishing some very necessary beds in the Liverpool Hospital for Women, Mrs. S. R. Graves has promised a donation of £25 as, also a double subscription if during the year the committee can raise the sum of £3,000 for the endowment of six beds £500 each.

THE committee of the Home Hospital, Fitzroy House, Fitzroy Square, have given the Lady Superintendent power to lessen the customary charges which are made for the rooms, under certain specified cases, where the patient's income is a very limited one.

THANKS to the sanitary precautions taken, the cholera present at Quimper in Brittany is greatly diminishing. During the past week three deaths only were recorded. A case of cholera is reported from Guernsey. The case, a fatal one, was that of a sailor hailing from Brittany.

THE 107th annual report of the National Truss Society shows that the work of the society continues to grow. No fewer than 2.662 surgical appliances have been supplied to poor persons during the year. We are glad to see an increase by 42 in the number of subscriptions. A handsome legacy has also been received, and donations have also advanced.

IN Birkenhead an agitation exists against the proposed new Fever Hospital, on the ground that needless expense is being incurred. But it should be borne in mind that the present hospital was condemned as far back as 1882, and the number of wards proved quite insufficient to meet the cases.

A BAZAAR in aid of the children's ward of the Northampton General Infirmary was held lately. The infirmary was erected on the occasion of the Queen's Jubilee in 1887. It is proposed to have a collection towards this fund, in which the schools are to be canvassed by selected school children.

FORTY-THREE thousand six hundred and forty-two cases have been treated at the London Temperance Hospital during the twenty years of its existence. At this hospital the visiting staff have full power to administer alcohol, but this treatment has only been resorted to seventeen times in the history of the establishment.

A GRATEFUL patient, a Mr. W. Lazenby, of Tennessea, who twelve months ago had been admitted as an in-patient to the Manchester Royal Infirmary, has become a regular subscriber to that institution, in recognition of the skilled attention he received from the nurses, since which time his wordly affairs have put him in a position to benefit the Infirmary.

THE twenty-six annual report of the Victoria Hospital for Children at Chelsea recorded a considerable advance in every direction. Last year 1,080 in-patients were received, and 17,075 out-patients. This hospital has now two convalescent homes attached to it; the older one being at Margate, and which has sheltered 3,000 children, and a new one, opened last year at Broadstairs.

### FIRST IMPRESSIONS OF BOOKS RECEIVED.

On the Application of Suitable Mechanism to a Case of Amputation of Both Hands. By F. Gustav Ernst, Orthopædic Mechanician to the National Orthoæpdic Hospital, &c.

There can be very few more crippled and helpless individuals than those who have lost altogether the two hands. Happily they are very rare. But a year or two ago such a case was referred to Mr. Ernst by Sir Joseph Lister, and the pamphlet before us contains many admirable photographs of the results of Mr. Ernst's skill. The success is so great that the patient can eat, drink, and be merry, and is no longer dependent upon others except in a minor degree. The photographs show the arms in many positions, and enables the reader to form a good opinion of the excellent result produced by Mr. Ernst. One must be a mechanician to understand and value aright Mr. Ernst's ingenuity and skill, and the better qualified one is to judge of his work the more highly will it be appreciated.

The Treatment and Disinfection of Scarlet Fever by Antiseptic Inunction. By J. Brendon Curgenven, M.R.C.S., L.S.A.

Mr. Curgenven maintains that the poison of scarlatina is only generated in the system during the first six days of the illness, and that the spores are then deposited in the epidermis. He is opposed to collecting cases of scarlatina together, as in that way patients continue to inhale the poison, and carry it about long after they have ceased to generate it. He advocates inunction of the whole body with Eucalyptus Disinfectant from the onset of the disease, twice a day for three days, and once a day for seven days more. He regards the patient as then infection-free. He also gives the eucalyptus internally. This treatment is recommended as modifying the course of the disease, preventing complications, and the spread of infection.

### Medical Vacancies and Appointments.

### APPOINTMENTS.

A. L. Galabin, M.D., M.A., appointed Examiner in Obstetric Medicine at the University of London. A. K. Gale, L.R.C.P.Lond., M.R.C.S.Eng., appointed Medical Officer of Health for the Ecclesall-Bierlow Rural Sanitary District. J. A. Goodfellow, M.B., C.M.Glas., reappointed Medical Officer of Health for the Brampton and Walton Sanitary Districts. B. W. Gowring, M.R.C.S.Eng., L.R.C.P.Lond., appointed House Surgeon to the Radcliffe Infirmary, Oxford. J. Graham, M.B., C.M.Edin., re-appointed Medical Officer for the No. 2 District of the Cockermouth Union. W. D. Halliburton, M.D., B.Sc., F.P.S., appointed Examiner in Physiology at the University of London. A. A. Hamilton, L.R.C.P., L.R.C.S.Edin., reappointed Medical Officer of Health for Crowle. T. H. Hitchins, M.R.C.S.Eng., L.S.A., reappointed Medical Officer of Health for the Shipton-on-Stour Union. H. Horsfall, M.D.St.And., M.R.C.S.Eng., re-appointed Medical Officer of Health to the Bedale Rural Sanitary District. R. K. Howat, M.B., C.M., M.R.C.S., L.R.C.P., appointed Clinical Assistant to the National Hospital for Diseases of the Heart and Paralysis, Soho-square. H. G. Howse, M.S., M.B., appointed Examiner in Surgery at the University of London. A. E. P. Hughes, M.R.C.S.Eng., L.R.C.P.Lond., appointed Dispensary Surgeon to the Bradford Infirmary. J. M. Hunt, M.B., C.M.Glasg., appointed Laryngologist to the Liverpool Royal Infirmary. W. H. Iddon, M.B.Lond., M.R.C.S.Eng., appointed Medical Officer for the Birkdale District of the Ormskirk Union. T. W. Kyle, M.D.Q.U.I., appointed Medical Officer of Health for Ashby-de-la-Zouch. F. R. Lathbury, L.S.A., appointed Medical Officer for the Sarratt District of the Watford Union. D. J. Lawson, M.D.Edin., appointed Medical Officer of Health to the Portland Sanitary District of the Weymouth Union. J. R. Levack, M.B., C.M. Aberd., appointed *pro tem.* Medical Officer to the Royal Infirmary and Eye Institution, Aberdeen. J. B. Littlejohn, M.B.Glasg., appointed Medical Officer for the Munslow District of the Ludlow Union. P. R. Littleton, M.R.C.S.Eng., reappointed Medical Officer of Health for the Ashbourne Urban Sanitary District. J. O. Littlewood, L.R.C.P.Lond., M.R.C.S.Eng., reappointed Medical Officer of Health to the Mansfield Rural Sanitary District. W. Livesay, M.D.Edin., appointed Public Vaccinator for the Sudbury District of the Uttoxeter Union. G. H. Longton, M.R.C.S., L.R.C.P., appointed Assistant House Accoucheur to King's College Hospital. R. C. Lucas, M.B., B.S., appointed Examiner in Anatomy at the University of London. A. P. Luff, M.D., B.Sc., appointed Examiner in Forensic Medicine at the University of London. G. Lumsden, M.B., C.M.Glasg., reappointed Medical Officer of Health to the Pateley Bridge Rural Sanitary District. T. F. Macdonald, M.B., C.M.Edin., appointed Resident Physician to the Royal Hospital for Sick Children, Edinburgh. A. A. Maclennan, M.B., C.M. Aberd., appointed Medical Officer of Health for the Burgh of Shetland. J. D Mann, M.D., appointed Examiner in Forensic Medicine at the University of London. J. R. Marshall, M.B., C.M.Glasg., appointed Medical Officer of Health for Bo'ness. A. E. Merewether, M.R.C.S.Eng., L.R.C.P., appointed Assistant Medical Officer to the Wandsworth Provident Dispensary. P. A. Nightingale, M.B., C.M., appointed Physician to His Highness the Sultan of Johore. T. A. Papillon, F.R.C.S.Edin., appointed House Surgeon to the Wolverhampton Eye Infirmary. J. F. Payne, M.D., B.Sc., appointed Examiner in the Practice of Medicine at the University of London. G. Porter, M.B., C.M.Edin., appointed Assistant House Surgeon to the General Infirmary at Gloucester and the Gloucestershire Eye Institution. L. Powne, M.R.C.S.Eng., reappointed Medical Officer for Newton Saint Cyres and Shobrooke Districts of the Crediton Union. Arthur Purkiss, M.D., C.M.Aberd., M.R.C.S.Eng., appointed Medical Officer and Public Vaccinator for the Wolston District of the Rugby Union. J. Richardson, L.R.C.P., L.R.C.S.Edin., appointed Junior

### MEDICAL APPOINTMENTS.

### LEICESTER INFIRMARY.

HENRY DOBBIN, Secretary.

Brompton, May 18th, 1893. (1,043)

### ROYAL VICTORIA HOSPITAL, Bournemouth.

At their QUARTERLY MEETING in July the Committee propose to ESTABLISH an OPHTHALMIC DEPARTMENT, and invite APPLICATIONS for the POST of OPHTHALMIC SURGEON.

Candidates are requested to send their applications, with testimonials, to the SECRETARY on or before June 1st. (1,000)

### ROYAL VICTORIA HOSPITAL, Bournemouth.

### ROYAL INFIRMARY, BRISTOL.

There is a VACANCY for an HONORARY ASSISTANT PHYSICIAN.

Applications and testimonials, by JUNE 10th, to the undersigned, who can forward a list of the Committee of Election.

J. F. SHEKLETON,

(862) Secretary and House Governor.

### ROYAL FREE HOSPITAL, Gray's Inn Road.

**MEDICAL VACANCIES AND APPOINTMENTS**—(*continued*)

House Surgeon to the Bradford Infirmary. H. L. Roberts, M.B., C.M.Edin., appointed Dermatologist to the Liverpool Royal Infirmary. E. S. Robinson, L.R.C.P.Lond., M.R.C.P.Eng., appointed Medical Officer of Health to the Stourport Local Board. W. Stirling, M.D., D.Sc., C.M., appointed Examiner in Physiology at the University of London. G. M. Swinhoe, L.R.C.P.Lond., M.R.C.S.Eng., reappointed Medical Officer of Health to the New Swindon Local Board. D. L. Thomas, M.R.C.S., L.R.C.P., L.S.A., appointed Clinical Assistant to the National Hospital for Diseases of the Heart and Paralysis, Soho Square. H. H. Tidswell, M.R.C.S.Eng., L.R.C.P.Lond., appointed Honorary Medical Officer to the Seaside Home, Whitby. N. J. C. Tirard, M.D., appointed Examiner in Materia Medica and Pharmaceutical Chemistry at the University of London. W. W. Walker, B.A., M.B., B.C.Cantab., appointed House Physician to the Great Northern Central Hospital, Holloway, N. D. C. Watson, M.B., C.M.Edin., appointed Resident Physician to the Royal Hospital for Sick Children, Edinburgh. W. Wyllie, M.D.Glasg., M.B., C.M., reappointed Medical Officer of Health for the Kirkby Lonsdale Urban Sanitary District. R. T. Bakewell, M.R.C.S. Eng., L.R.C.P. Lond., appointed Junior Resident Medical Officer to the Royal Free Hospital, Gray's Inn Road.

### VACANCIES.

The following vacancies are announced this week, the amount of salary in each case being given after the name of the institution:

**Resident Medical Officers.**

Central London Ophthalmic Hospital, 238A, Gray's Inn Road, W.C. House-Surgeon. Applications to the Secretary by June 6th.

Children's Hospital, Steelhouse Lane, Birmingham. Resident Medical Officer. Salary £70 per annum, with board, washing, and attendance; and Resident Surgical Officer. Salary £50 per annum, with board, washing, and attendance. Applications to the Secretary by May 31st.

Coventry and Warwickshire Hospital, 10, Hay Lane, Coventry. House-Surgeon, doubly qualified. Salary £100 per annum, with board, rooms, and residence. Assistant House-Surgeon. Honorarium of £15, with board, rooms, and attendance. Applications to Arthur Seymour, Secretary, by May 31st.

East London Hospital for Children, Glamis Road, Shadwell, E. House-Surgeon. Board and lodging provided. Applications to the Secretary, by June 7th.

Leicester Infirmary. Assistant House-Surgeon. Salary £80 per annum, with board, apartments, and washing. Applications to the Secretary, 24, Friar Lane, Leicester, by May 27th.

North-Eastern Hospital for Children, Hackney Road, N.E. Junior House-Surgeon, doubly qualified. Appointment for six months. Salary at the rate of £60 per annum. Applications to Alfred Nixon, Secretary, 27, Clement's Lane, E.C., by June 10th.

Royal Victoria Hospital, Bournemouth. House Surgeon Secretary. Salary £100 per annum, with board. Applications to the Chairman of the Committee by July 1st.

Rubery Hill Asylum, near Bromsgrove. Clinical Assistant. Applications to the Medical Superintendent.

Wolverhampton and Staffordshire General Hospital, Wolverhampton. Resident Assistant. Board, lodging, and washing provided. Applications inscribed "Applications for Resident Assistant" to the Chairman of the Medical Committee by June 1st.

**Non-Resident Medical Officers.**

Chelsea Hospital for Women, Fulham Road, S.W. Physician to out-patients. Applications (on forms to be obtained) to A. C. Davis, Secretary, by June 5th.

Hospital for Consumption and Diseases of the Chest, Brompton. Assistant Physician. Applications to Henry Dobbin, Secretary, by June 7th.

Mason College, Birmingham. Professor of Pathology. Applications to Geo. H. Morley, Secretary, by June 3rd.

Royal Infirmary, Bristol. Honorary Assistant Physician. Applications and testimonials to J. F. Shekleton, Secretary and House Governor (who can forward a list of the Committee of Election) by June 10th.

Royal Victoria Hospital, Bournemouth. Ophthalmic Surgeon. Applications to the Chairman of the Committee by July 1st.

St. George's Hospital, S.W. Visiting Apothecary. Applications to the Secretary by May 27th.

Stockton-on-Tees Hospital and Dispensary. House-Surgeon, doubly qualified. Salary £200 per annum. Applications to Thomas Bradley, Secretary, by May 30th.

Sunderland Infirmary. Honorary Medical Officer to the Convalescent House at Heatherdene, Harrogate, doubly qualified. Applications to the Chairman of the Medical Board by June 6th.

University College, Bristol. Professor and two Demonstrators of Anatomy. Applications to James Rafter, Secretary, by May 23rd.

